# *CFTR* Cooperative *Cis*-Regulatory Elements in Intestinal Cells

**DOI:** 10.3390/ijms22052599

**Published:** 2021-03-05

**Authors:** Mégane Collobert, Ozvan Bocher, Anaïs Le Nabec, Emmanuelle Génin, Claude Férec, Stéphanie Moisan

**Affiliations:** 1 Univ. Brest, Inserm, EFS, UMR 1078, GGB, F-29200 Brest, France; bocherozvan@gmail.com (O.B.); lenabec.anais@gmail.com (A.L.N.); emmanuelle.genin@inserm.fr (E.G.); claude.ferec@univ-brest.fr (C.F.); 2 Department of Molecular Genetics and Reproduction Biology, CHRU Brest, F-29200 Brest, France

**Keywords:** *cis*-ruption disorders, *CFTR*, regulation, transcription factors, 3D structure

## Abstract

About 8% of the human genome is covered with candidate *cis*-regulatory elements (cCREs). Disruptions of CREs, described as “*cis*-ruptions” have been identified as being involved in various genetic diseases. Thanks to the development of chromatin conformation study techniques, several long-range cystic fibrosis transmembrane conductance regulator (*CFTR*) regulatory elements were identified, but the regulatory mechanisms of the *CFTR* gene have yet to be fully elucidated. The aim of this work is to improve our knowledge of the *CFTR* gene regulation, and to identity factors that could impact the *CFTR* gene expression, and potentially account for the variability of the clinical presentation of cystic fibrosis as well as *CFTR*-related disorders. Here, we apply the robust GWAS3D score to determine which of the *CFTR* introns could be involved in gene regulation. This approach highlights four particular *CFTR* introns of interest. Using reporter gene constructs in intestinal cells, we show that two new introns display strong cooperative effects in intestinal cells. Chromatin immunoprecipitation analyses further demonstrate fixation of transcription factors network. These results provide new insights into our understanding of the *CFTR* gene regulation and allow us to suggest a 3D *CFTR* locus structure in intestinal cells. A better understand of regulation mechanisms of the *CFTR* gene could elucidate cases of patients where the phenotype is not yet explained by the genotype. This would thus help in better diagnosis and therefore better management. These *cis*-acting regions may be a therapeutic challenge that could lead to the development of specific molecules capable of modulating gene expression in the future.

## 1. Introduction

Genomic architecture has been far better investigated and understood in the last one or two decades. Many studies have focused on the role of non-coding regions and on genetic variants they contain, opening up new research possibilities. It is established that 7.9% of the human genome is covered with candidate *cis*-regulatory elements (cCREs) [1]. Anomalies of *cis*-regulatory elements (CREs) at distance from a gene have been identified as being involved in various genetic diseases [2,3]. These anomalies have an impact on *cis*-regulatory sequences (by deletions [4], translocation [5], mutations [6,7] or duplications [8]). They can also affect the organization of topologically associated domains (TADs) [9] when they are located in boundaries regions, and alter the CTCF (CCCTC-binding factor)/cohesin complex fixation [4,10]. The terms “*cis*-ruption disorder” and “enhanceropathies” have been proposed, to describe pathologies whose origin comes from dysfunction of CREs [11].

Cystic Fibrosis (CF) is an autosomal recessive disease caused by mutations in the gene encoding the *cystic fibrosis transmembrane conductance regulator* (*CFTR*) [12]. This gene has been extensively studied for more than 30 years throughout the world consortium [13]. Over 2100 variants have been identified in this gene (Cystic Fibrosis Mutations Database) causing CF or *CFTR*-related disorder (*CFTR*-RD) such as congenital bilateral absence of vas deferens CBAVD, pancreatitis, or bronchiectasis [14,15]. The gene encodes the CFTR protein which is found in the apical plasma membrane of airway, intestinal, epididymal and exocrine epithelial cells.

Most of the work on the molecular basis of CF has been focused on the identification of variations located in the *CFTR* coding regions, and understanding the high degree of variability in disease severity, complications as well as survival observed among CF. A part of this phenotypic variability could be explained by allelic heterogeneity in the *CFTR* gene. One of the best illustrations is the correlation between *CFTR* genotype and pancreatic disease severity [16]. The identification of modifiers genes has brought new insights in this field [17,18], but there are still some unexplained phenotypic variations.

Despite this large amount of work, the regulation of the *CFTR* gene itself, which is critical and not completely understood, could probably explain at least a part of this phenotypic variability. On a large scale, the identification of CREs and a better understanding of the *CFTR* gene regulatory mechanisms could explain the phenotypic variability, and be promising therapeutic targets in cystic fibrosis and its related disorders.

The *CFTR* gene has a tissue-specific expression, with variable *CFTR* transcript levels according to cell types [19,20,21,22]. Regulatory elements described in the promoter [23,24,25,26], cannot alone explain this complex tissue-specific regulation. Since the development of chromatin conformation study techniques [27,28,29], several long-range regulatory elements have been identified in DNase hypersensitive sites (DHSs) [30,31,32,33,34]. *CFTR* is one of the first genes for which critical CREs for its accurate regulation have been identified at distance from its promoter [23,24,25,26]. A *CFTR* sub-TAD was further highlighted by the recruitment of CTCF factors at the opposite edges of the locus: at−80.1 kb and +48.9 kb relative to the *CFTR* gene, common to all cell types [33,35,36,37]. The formation of a DNA loop ensures distant CREs to interact at proximity of the *CFTR* promoter [34,35,36,37] and establishes a 3D regulation organization of the *CFTR* locus.

The objective of this work is to improve our knowledge of the fine regulation of the *CFTR* gene, and to identify factors that could impact the *CFTR* gene expression. All of this to provide future opportunities for therapeutic targeting to improve the management and quality of life of patients with cystic fibrosis or *CFTR*-RD.

In order to identify all intronic cCREs, we used the robust GWAS3D score [38] cCREs and identified four *CFTR* introns that seem to be involved in gene regulation. To this extent, we have been able to confirm two previously *CFTR* intestinal CREs already described in introns 1 and 12. Interestingly, we further demonstrate the involvement of two new cCREs of introns 24 and 26 in the regulation of the *CFTR* locus. Thus, we show strong cooperative enhancer effects between these CREs on the *CFTR* promoter activity. Finally, we also highlight the binding of important transcription factors (TFs) (such as HNF1α, p300, FOXA1/2, CDX2 and TCF4) to these new *CFTR* CREs. Building on these results, we propose a completely new 3D regulation model of the *CFTR* locus in intestinal cells.

## 2. Results

### 2.1. GWAS3D Score Predicts Four Regions as Candidate Cis-Regulatory Elements of the CFTR Gene

To determine which of the *CFTR* introns could be involved in gene regulation, we used the GWAS3D score proposed by Li et al. [38]. This score is based on different functional information of regulation, such as Dnase-seq, TF ChIP-Seq, histone modifications and 5C data [39,40], and a higher score is predictive of a more important functional impact of the variant. We compared the distribution of the scores of variants in the 1000 Genomes project [41] between all *CFTR* introns with the hypothesis that an intron showing more variants with high GWAS3D scores has a stronger functional effect.

**Figure 1:**
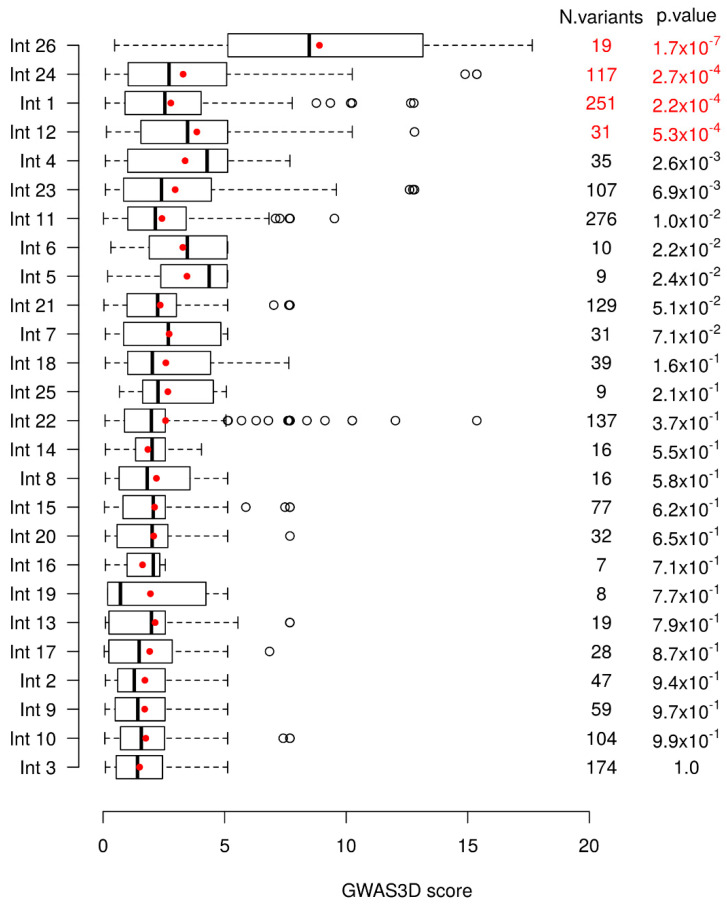
Variants distribution of the GWAS3D scores in cystic fibrosis transmembrane conductance regulator (*CFTR*) introns. Boxplots of the GWAS3D scores of the 1000 Genomes Phase 3 variants located in the different introns are shown with the mean score indicated by a red point. Introns are ranked based on the *p*-value of the Wilcoxon test that compares the GWAS3D scores of their variants against those of the variants located in all other introns sequentially. These *p*-values and the number of variants (N variants) in each intron are provided in the last two columns. *p*-values in red are those significant at the 5% level after Bonferroni correction.

A global significant difference of GWAS3D scores between introns is observed (Kruskal–Wallis test with 25 degrees of freedom, *p*-value = 3.25 × 10^−15^). To further see which intron(s) differed from others, we compared each intron to all others combined using a unilateral Wilcoxon test. We then removed the most significant intron, and repeated the analysis using a sequential procedure until no more introns were significant at the Bonferroni correction. Based on this, we tested each intronic region for an enrichment in variants found in 1000 Genomes with high GWAS3D scores. We found four introns significantly different from the other ones (Figure 1). Intron 26 was the most significant one, followed by intron 24, intron 1, and finally intron 12.

**Figure 2:**
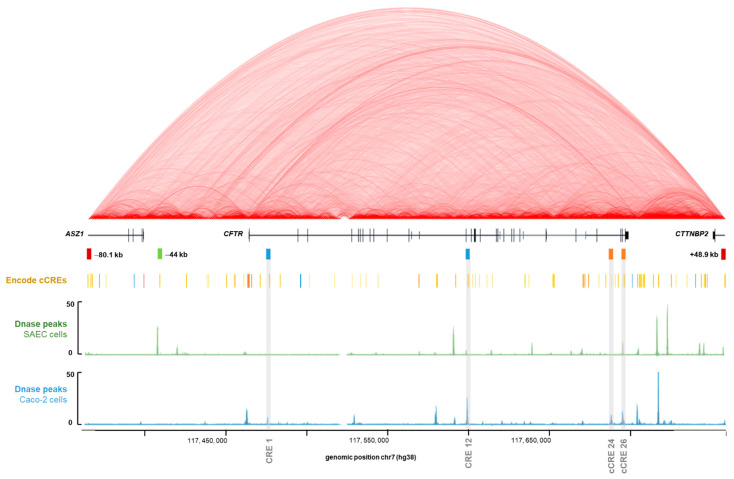
Linear schematic representation of the *CFTR* genomic study region. Visualization of regulation data of the ENCODE Consortium project using UCSC genome browser (http://genome.ucsc.edu/ENCODE, accessed on 20 May 2020, [45]) with our four *CFTR* intronic regions of interest, predicted by the GWAS3D score. Boundaries of the *CFTR* sub-topologically associated domains (TAD) are already known, with insulators at −80.1 kb and +48.9 kb, represented by red rectangles. Introns 1 and 12 *cis* regulatory elements (CREs) have already been described as two main *cis*-regulatory cooperative elements implicated in *CFTR* gene regulation in intestinal cells, represented by blue rectangles. The −44 kb CRE is one of the most important enhancers in airway cells, represented by a green rectangle. Alignment of Hi-C data track corresponds to heatmaps of chromatin folding from human Embryonic Stem Cell line H1 (H1-hESC) indicating interactions detected within the genome in this cell type, and the degree of interaction is indicated by a more intense red color. This Hi-C data track is represented in red arc mode with a score maximum of 11 and a 1 kb resolution. According to ENCODE Consortium, our regions are considered as distal enhancer-like signature, represented by yellow rectangles on the line “ENCODE cCREs”. Alignment of ENCODE Registry of cCREs track to highlight the position of cCREs in the human genome from ENCODE Consortium, over the schematic linear representation of the region (http://genome.ucsc.edu/ENCODE, accessed on 20 May 2020) [42,45,46]. Alignment of DNAse I hypersensitivity data (DNase hypersensitive sites (DHSs) peaks) from SAEC was represented by a green schematic linear. Alignment of DHSs peaks from Caco-2 cells was represented by a blue schematic linear [46,47]. DHS peaks identify open chromatin sites. cCREs: candidate *cis*-regulatory elements.

The Encyclopedia of DNA Elements (ENCODE) Project, aims to identify all the functional elements of the human genome, and to date has identified 926,535 cCREs across the entire human genome [42]. Chromatin opening has a positive role in gene expression [43], and it is notably at the level of the DHS regions that cCREs can be identified [44]. Indeed, we know that tissue-specific cCREs have been described from intronic DHSs of the *CFTR* gene. When looking at our four intronic regions of interest in the ENCODE database, viewed via UCSC, we confirm the presence of DHS signals depending on the cellular type (Figure 2). The chromatin conformation High throughput 3C (Hi-C) data available via UCSC show strong interactions within the *CFTR* subTAD, demonstrating important regulatory activities for *CFTR* gene expression (Figure 2). According to ENCODE, our regions are considered as cCREs with distal enhancer-like signatures (dELS) (Figure 2). Our previously described analysis of GWAS3D scores to predict the involvement of intron potentials in gene regulation allows us to confirm its robustness, as we highlight the two most important intronic enhancers in *CFTR* gene regulation, as well as two new introns.

Regions of intron 1 at 185 + 10 kb and intron 12 at 1811 + 0.8 kb (intron 11 of the previous nomenclature) have already been described as two main *cis*-regulatory cooperative elements implicated in *CFTR* gene regulation in intestinal cells [24,25,31]. Studies have shown a cooperation between intestinal cells specific TFs, such as hepatocyte nuclear factor 1 α (HNF1α), caudal type homeobox 2 (CDX2), E1A binding protein p300 (p300) and forkhead box protein A1/A2 (FOXA1/A2), which bind to CREs in introns 1 and 12 [31,32,48,49,50,51]. In addition, the histone, T-cell factor 4 (TCF4) binds the intron 1 in intestinal cells [49], and is described as an important TF in intestinal cells function and differentiation [52]. Thus, all these TFs were found to be essential for maintaining high levels of *CFTR* expression in Caco-2 cells [50].

In contrast, introns 24 and 26 have been little studied to date. A DHS at 4374 + 1.3 kb (chr7:117, 665, 699-117, 666, 713; hg38) in the intron 26 of the *CFTR* gene (intron 23 of the previous nomenclature) was identified in pulmonary, intestinal and epididymis cells [31]. This DHS had a very modest enhancer activity, not statistically significant, with the *CFTR* promoter. However, in combination with two other specific pulmonary cells, DHS at -35 kb and −3.4 kb of the *CFTR* gene, this intron 26 DHS increased by 60-fold the promoter activity in pulmonary 16HBE14o-cells [53]. FOXA1 and FOXA2 enrichment were identified on this DHS in intestinal cells Caco-2 [48,50]. Inside the intron 24 of the *CFTR* gene (intron 21 of the previous nomenclature), a DHS at 4095 + 7.2 kb (chr7:117, 659, 365–117, 660, 175; hg38) was identified [26], with a very low enhancer activity [54] since no test had been carried out in this cCRE region.

### 2.2. Intestinal Specific Activity of Intron 24 CRE

Among the four introns highlighted by the GWAS3D scores, introns 1 and 12 were described as specific enhancers in intestinal cells [24,25,31] and only as weak enhancers in pulmonary cells [24,53]. The intron 26 cCRE was described as low enhancer in different cells-types (pulmonary, intestinal and epididymis). To better describe the intron 24 region, we sought to determine its specific tissue expression by luciferase reporter gene.

To characterize the regulatory activity of intron 24 cCRE on the *CFTR* gene, gene reporter assays were conducted in pulmonary 16HBE14o- and intestinal Caco-2 cell lines mainly used in *CFTR cis*-regulatory enhancers studies [55]. Among all the cell types expressing the *CFTR* gene, digestive system cells were found to be the ones showing the strongest expression [56].

Candidate *cis*-regulatory elements were inserted in the BamHI specific enhancer insertions site of the pGL3-Basic vector in which 787 bp of the *CFTR* minimal promoter (P*_CFTR_*) drives luciferase expression. Constructs were evaluated for firefly luciferase expression following transient transfection into cell lines. Luciferase expression values were reported in comparison with the P*_CFTR_* luciferase expression alone.

**Figure 3:**
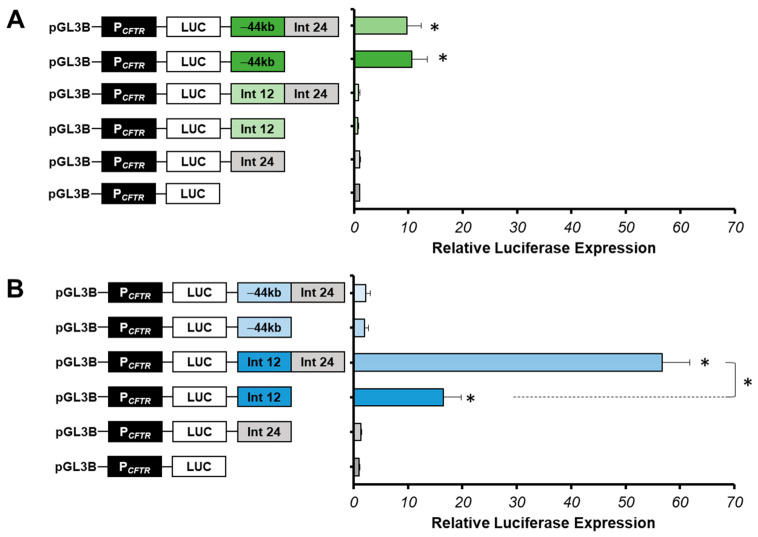
Cell-type specificity of the intron 24 cCRE. (**A**) Luciferase reporter constructs with the *CFTR* promoter (P*_CFTR_*; 787 bp) and *cis*-regulatory elements combined or alone (Intron 24:Int24; Intron 12:Int12; −44 kb) were transfected in pulmonary 16HBE14o-cells. Intron 12 enhancer is a positive control. (**B**) Luciferase reporter constructs with the *CFTR* promoter (P*_CFTR_*; 787 bp) and *cis*-regulatory elements combined or alone (Int24; Int12; −44 kb) were transfected in intestinal Caco-2 cells. Enhancer at −44 kb of the *CFTR* gene is a positive control in pulmonary 16HBE14o- cells, and a negative control in intestinal Caco-2 cells. Luciferase expression values are reported at β-galactosidase expression values (control of transfections). Error bars represent the standard error of the mean (SEM; *n* = 9), * *p* < 0.002, using unpaired *t*-tests.

We show that the luciferase expression does not increase with the intron 24 cCRE in 16HBE14o-cells compared with the P*_CFTR_* luciferase expression, suggesting that this region does not have an enhancer activity in pulmonary cells (Figure 3A). However, in Caco-2 cells, a low increase in luciferase expression (1.32-fold) is observed compared to the P*_CFTR_* luciferase expression alone, suggesting a low enhancer activity in intestinal cells (Figure 3B).

Several studies have shown that combinations of enhancers increase the *CFTR* promoter activity compared to the region alone [31,33,34,54]. Indeed, the intron 24 cCRE could have an enhancer activity in cooperation with other tissue-specific enhancers. Several combinations of this region with strong specific enhancers described in pulmonary 16HBE14o- and in intestinal Caco-2 cells have been performed.

A combination between the intron 24 cCRE and the −44 kb CRE was produced in 16HBE14o-cells. The −44 kb CRE of the *CFTR* gene is one of the most important enhancers in airway cells (chr7:117, 434, 794–117, 436, 591, hg38) [53,57]. In this study, the −44 kb enhancer served as a positive control in 16HBE14o- lung cells and as a negative control in Caco-2 intestinal cells. Activity tests have showed that the luciferase expression does not increase with the combination −44 kb-Int24 (9.7-fold) compared to the luciferase expression of the −44 kb CRE alone (10.6-fold) (Figure 3A). This suggests that intron 24 cCRE does not act with the −44 kb CRE in 16HBE14o-cells.

We looked at enhancer activity of the intron 24 cCRE in Caco-2 cells in combination with the intron 12 CRE, which is the strongest enhancer specific to intestinal cells [31]. The combination of these two DHS of introns 12 and 24 induces a strong cooperative effect, increasing the luciferase expression by 57-fold, around six times the effect of the intron 12 enhancer alone (Figure 3B). Thus, intron 24 cCRE has a strong enhancer activity only in cooperation with the intron 12 enhancer in intestinal cells Caco-2.

**Figure 4:**
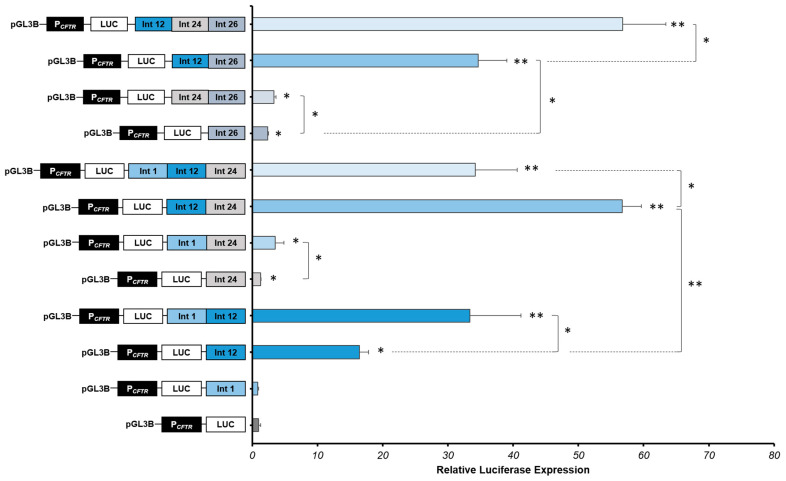
Strong cooperative effects between *CFTR* introns CREs in intestinal cells. Luciferase reporter constructs with the *CFTR* promoter (P*_CFTR_*; 787 bp) and *cis*-regulatory elements combined or alone (DHS Intron 1:Int 1; DHS Intron 12:Int 12; DHS Intron 24:Int 24; DHS Intron 26:Int 26) were transfected in intestinal cells Caco-2. Enhancers of introns 1 and 12 of the *CFTR* gene are positive controls. Luciferase expression values were reported at β-galactosidase expression values (control of transfections). Error bars represent the standard error of the mean (SEM; *n* = 9), * *p* < 0.002, ** *p* < 10^−10^ using unpaired t-tests.

Moreover, we confirm that intron 26 cCRE alone slightly increases the P*_CFTR_* activity in Caco-2 cells (2.36-fold) (Figure 4) [53].

In view of the results from the study of 1000 Genomes GWAS3D scores and activity tests carried out in 16HBE14o- pulmonary and Caco-2 intestinal cells, we decided to continue the study of our cCREs of interest (enhancers of introns 1, 12, 24 and 26) in their preferential cell-type: Caco-2 intestinal cells.

### 2.3. Strong Cooperative Effects between Relevant Introns CREs in Intestinal Cells

Introns 24 and 26 DHS have been little studied because of their low enhancer activity obtained by luciferase assays. We wanted to further investigate the activity of these two regions in the Caco-2 intestinal cell line and especially in combination. To characterize the regulatory activity of introns 24 and 26 cCREs of the *CFTR* gene in combination with other intestinal specific CREs, activity tests have been managed in Caco-2 cell lines. Several combinations of these cCREs have been performed, notably with introns 1 and 12. Results obtained by the GWAS3D score suggest that these four intronic regions may act together in the *CFTR* gene regulation. For this, we decided to test these four regions of interest in combination by luciferase gene reporter.

We confirm that intron 1 CRE alone has no effect on the *CFTR* promoter activity, and we confirm the high activity of the intron 12 CRE in Caco-2 cells, increasing 16-fold the *CFTR* promoter activity (Figure 4).

We show that the combination of introns 1 and 12 enhancers increases significantly the *CFTR* promoter activity by 33-fold (Figure 4), while the combination of introns 1 and 24 cCREs increase the *CFTR* promoter activity in Caco-2 cells by 3.53-fold (Figure 4). These first results enable us to highlight that the intron 1 enhancer acts with the intron 12 enhancer, but also, not so much but still significant, with the intron 24 cCRE. Moreover, the combination of introns 12 and 24 enhancers induces a strong cooperative effect, increasing the luciferase expression by 57-fold, around six times the effect of the intron 12 enhancer alone (Figure 4) and almost twice that of intron 1-12 cooperation. Combination of introns 1, 12 and 24 enhancers induces a strong effect, increasing significantly the luciferase activity by 34-fold, a similar level that the intron 1-12 combination, but less than with combination of introns 12 and 24 (Figure 4). Thus, intron 24 cCRE has a real enhancer activity but only in cooperation in intestinal cells Caco-2.

Thereafter, we have evaluated the effect of the intron 26 cCRE in combination with introns 12 and/or 24 enhancers in Caco-2 cells. The luciferase expression is slightly but significantly increased in combination with the intron 24 cCRE, increasing 3.32-fold the *CFTR* promoter activity. However, in combination with the intron 12 enhancer, the luciferase is strongly expressed, increasing the *CFTR* promoter activity by 35-fold. The *CFTR* promoter activity is even greater with the combination of these three enhancers (introns 12, 24 and 26), increasing the activity in Caco-2 cells by 57-fold.

The strong cooperative effect of these CREs induces a robust enhancement of the *CFTR* promoter activity in Caco-2 intestinal cells. Introns 24 and 26 enhancers have an important role in the regulation of the *CFTR* locus, combining with introns 1 and 12 CREs.

### 2.4. Intestinal Cell-Specific Transcription Factors Bind on Introns 24 and 26 CREs

To better understand how these enhancers may act together within the *CFTR* sub-TAD, we looked to determine whether intestinal cell-specific TFs bind to introns 24 and 26 CREs. Chromatin immunoprecipitations (ChIP) have been realized from 4.10^6^ Caco-2 intestinal cells per immunoprecipitation, in order to investigate HNF1α, p300, FOXA1/2, TCF4 and CDX2 bindings to introns 24 and 26 enhancers. These factors were previously described enriched on introns 1 and 12 [31,48,49,50,51]. They form a protein network and are important in intestinal cells’ specific regulation [58].

In silico predictive analyses of potential transcription factor binding sites (TFBSs) were performed via the ConTra v3 web server. ConTra v3 allows visualization and exploration of predicted TFBSs in the whole genome, and to assign an affinity score of TFBSs [59]. We used predicted TFBSs directly, rather than relying on data from ChIP-seq, whose sites are too large. We have therefore designed different pairs of specific primers for the targeted TFBSs.

**Figure 5:**
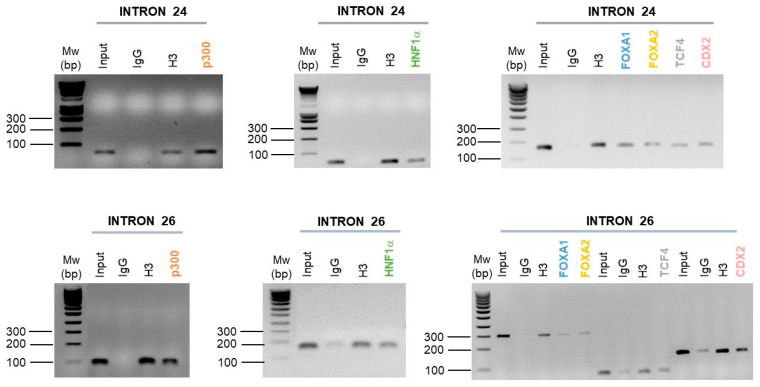
Enrichment of intestinal cell-specific transcription factors on introns 24 and 26 *CFTR* CREs. Enrichment of p300, HNF1α, FOXA1, FOXA2, TCF4 and CDX2 transcription factors have been performed by chromatin immunoprecipitation in Caco-2 intestinal cells. Targeted transcription factors have been tested on the intron 24 and the intron 26 of the *CFTR* gene. The enrichment has been analyzed by polymerase chain reaction and agarose gel (1.5%).

Following the ChIP-PCR carried out, and in comparison to the negative control (IgG), we show an enrichment of HNF1α, FOXA1, FOXA2, CDX2 and p300 in introns 24 and 26 CREs of the *CFTR* gene (Figure 5). However, the TCF4 factor is only enriched on the intron 24 CRE (Figure 5).

These ChIP analyses have thus highlighted the binding of a large network of TFs described as important in the *CFTR* gene regulation in intestinal cells.

## 3. Discussion

The *CFTR* gene was studied for many years, resulting in the identification of a large number of variants, more than 2100. Despite this, cases still remain with an incomplete genotype, mainly *CFTR*-RD, and patients with extreme phenotypes, such as early or late bacterial colonization or defaults of maximum expiratory volume per second. All these data highlight the importance of further study on the regulation of *CFTR* gene, to underline and understand the role and implication of *CFTR* cCREs in specific cell types, in order to illustrate their potential roles in CF and/or *CFTR*-RD. Alteration of cCREs could therefore elucidate these unexplained cases of CF or *CFTR*-RD. Two variants in the 5’ UTR have already been identified as regulatory mutations, decreasing the *CFTR* gene expression [60,61]. In addition, a better understanding of *CFTR* gene regulation offers opportunity for new therapeutic strategies for patients and thus to improve their management and quality of life.

To investigate this question, we used the GWAS3D score [38] to identify introns enriched in important functional variants in the *CFTR* gene regulation in the general population. This score is based on different functional information such as DNA-seq, TF ChIP-Seq, histone modifications and 5C data [40], and enables us to rank variants according to their predicted functional impact. We identify four introns, introns 26, 24, 1 and 12, in order of importance (Figure 1) showing GWAS3D scores significantly higher than the other introns. DHSs in introns 1 and 12 have already been well-documented as *cis*-regulatory elements with enhancer activity on the *CFTR* gene, particularly in intestinal cells [31,37,62]. In contrast, DHSs in introns 24 and 26 identified, have been very little studied, especially the intron 24. The DHS at 4374 + 1.3 kb described in the intron 26 has been highlighted in pulmonary, epididymal and intestinal cells, with a low enhancer activity. The DHS at 4095 + 7.2 kb described in the intron 24 has been identified in intestinal cells, with a low enhancer activity [54], but no test has been continued with this DHS. Moreover, the database ENCODE project supports our GWAS3D score results, as it predicts our cCREs as distal enhancer-like signature (Figure 2).

By functional tests, we sought to determine the tissue-specific expression (pulmonary and/or intestinal) of the intron 24. We show that intron 24 cCREs have an enhancer activity specific for Caco-2 intestinal cells, as for introns 1, 12 and 26, which is why we decided to continue functional tests in this cell type. We tested, by luciferase reporter assay, introns 24 and 26 cCREs activities in Caco-2 intestinal cells and confirm that these DHSs have a low enhancer activity alone in intestinal cells. Strong cooperative effects have been highlighted with others specific CREs (Introns 1 and 12) in intestinal cells (Figure 4). We can confirm that introns 24 and 26 enhancers have an important role to increase the *CFTR* activity in intestinal cells by an enhancer activity in combination.

We observe that the combination of introns 12 and 24 enhancers increases significantly the *CFTR* promoter activity compared to the combination of introns 1 and 24 or introns 1, 12 and 24 enhancers. This can probably be explained by a competition between introns 1 and 24 enhancers to interact with the intron 12 enhancer in order to regulate the *CFTR* promoter.

HNF1α, P300, CDX2 and FOXA1/2 bind on introns 1 and 12 CREs [31,32,48,49,50,51], TCF4 factor binds also the intron 1 enhancer [49], and only enrichments of FOXA1/2 have been described on the intron 26 enhancer in intestinal cells [31,49,51,54]. These factors are important for the *CFTR* gene regulation in intestinal cells, particularly in the opening of the chromatin by FOXA1 and FOXA2 factors [51,52,59]. In this study, ChIP assays show enrichments of HNF1α, p300, FOXA1/2 and CDX2 on introns 24 and 26 CREs (Figure 5). The TCF4 factor is only enriched on the intron 24 enhancer (Figure 5). The TCF4 factor was described as important TF in intestinal cells regulation [52], but is probably not involved in the TFs network attached to the intron 26 enhancer. As described on introns 1 and 12 [51,52,59], the strong TFs network including HNF1α, p300, FOXA1/2 and CDX2 bind introns 24 and 26, supporting that this CREs may play an important role in the *CFTR* gene regulation via this wide network of TFs interaction.

Several chromatin conformation analyses using Chromosome Conformation Capture 3C [27], Circularized Chromosome Conformation Capture 4C [28] and Carbon Copy Chromosome Conformation Capture 5C [29] showed interactions between the *CFTR* gene promoter and several CREs in different cell types, such as intestinal, pulmonary or epididymal cells [31,34,37,62]. 4C analyses in intestinal Caco-2 cells showed interactions between the promoter and specific CREs, such as at −80.1 kb, −20.9 kb, introns 11 and 12, +6.8 kb, +15.6 kb and +48.9 kb. All these studies, discussed in the latest review by Ann Harris’s team, have made it possible to propose a 3D regulation model of different CREs identified in airway and intestinal cells [55], notably highlighting introns 1 and 12 CREs as the main regulatory actors via a specific network of FTs in intestinal cells.

Here, an innovative bioinformatics analysis using GWAS3D score, has permitted us to highlight four introns of interest, including two new intronic enhancers, as important in the *CFTR* locus regulation. The importance of these intronic regions has been confirmed by luciferase reporter assays as strong enhancers in combination in intestinal cells, playing a role in the *CFTR* gene regulation. ChIP analyses have permitted to support the role of these CREs in intestinal cells by the recruitment of specific TFs network, including HNF1α, p300, FOXA1/2 and CDX2. With these results, our hypothesis would be that introns 24 and 26 enhancers interact with the intron 12 CRE, which itself interacts with the promoter, in particular through an intestinal cell-specific TFs network.

**Figure 6:**
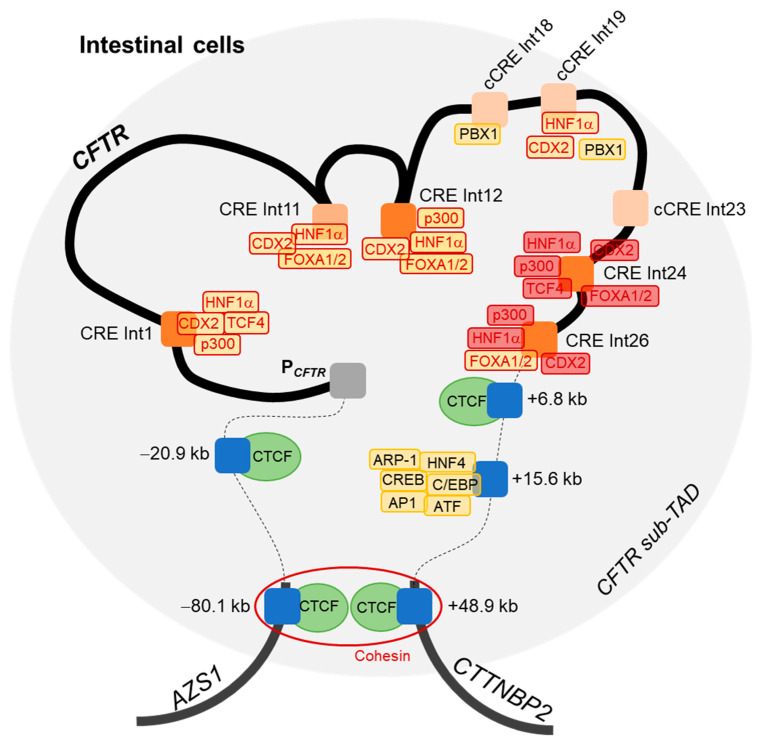
Model of three-dimensional regulation of the *CFTR* locus in intestinal cells. A DNA loop is formed by the recruitment of CTCF factors at −80.1 kb and +48.9 kb of the *CFTR* gene, in complex with cohesin (red ring), delineating boundaries of the topologically associated domain (TAD) of the *CFTR* locus (blue rectangles representing the barrier insulators and the red circle representing the cohesin). DNA loops and binding of transcription factors (TFs) (yellow blocks) allow the proximity of DNase hypersensitive sites (DHSs) present in introns (Int) and described as enhancers (orange blocks) with the promoter (P*_CFTR_*). Blue blocks represent insulators located upstream or downstream of the *CFTR* gene. Dark orange blocks correspond to our intronic regions of interest, whereas light orange blocks have been previously reported but not studied in this work. TFs with red contours correspond to factors of interest, such as HNF1α, p300, FOXA1, FOXA2, CDX2 and TCF4, which have been studied for their binding with introns 24 and 26 (block filled with red).

All these results lead us to propose an updated 3D model of the *CFTR* locus regulation in intestinal cells (Figure 6). Boundaries of the *CFTR* sub-TAD are already known, with insulators at −80.1 kb and +48.9 kb, which allow the formation of a DNA loop via the recruitment of CTCF factors in complex with cohesin [37]. As mentioned above, interactions between the *CFTR* promoter and CREs in introns 1 and 12 have been described, and with CREs at −20.9 kb, +6.8 kb, +15.6 kb of the *CFTR* gene. 

This study pinpoints that DHSs that have been little studied before, appear to be relevant in the *CFTR* gene regulation and permits the refinement of the understanding of CREs in the regulation of this gene. In the genome, CREs are important in the locus organization, in particular through the implementation of boundary barrier insulators of the sub-TADs, and within the locus to allow gene regulation through CREs interactions [62,63]. This suggests that variants in critical CREs could deregulate gene organization. Regulatory element dysfunctions have already been identified, known as *cis*-ruption disorders, particularly in developmental pathologies [11]. It is therefore clear that regulatory variants analysis in critical CREs of the *CFTR* gene should be investigated. For example, a deep study of the *CFTR* locus in 762 CF patients allowed the identification of such variants, notably in the −80.1 and −44 kb CREs of the *CFTR* gene [64]. No functional tests were conducted in this study.

All these analyses enabled us to better understand the *CFTR* gene regulation, its tissue-specific expression, and ultimately to modulate it. These *cis*-acting regions may be a therapeutic challenge that could lead to the development of specific molecules capable of modulating gene expression in the future. Moreover, a better understanding of regulatory mechanisms of this gene responsible for cystic fibrosis could elucidate cases of patients where the phenotype is not yet explained by the genotype. This would thus help in better diagnosis and therefore better management.

## 4. Materials and Methods

### 4.1. GWAS3D Score

To determine which of the *CFTR* introns could be involved in gene regulation, we used the GWAS3D score proposed by Li et al. [38]. This score is based on different functional information such as DNA-seq, TF ChIP-Seq, histone modifications and 5C data (see Nishisaki and Boyle., 2017 for a comparison of several commonly used scores) [39]. GWAS3D scores of 1000 Genomes Phase 3 variants located in the different introns were obtained from regBase [40] (regBase_Common V1.0 downloaded from https://github.com/mulinlab/regBase, accessed on 7 October 2019). To test if there was a global difference of GWAS3D scores distribution between the different introns, we used a Kruskal–Wallis test. To further assess which introns differ most from the others, we used the following sequential procedure: (i) for each intron, we tested if the GWAS3D scores of its variants were higher than the GWAS3D scores of all other variants using unilateral Wilcoxon tests, (ii) we then removed from the analysis the most significant intron and assigned it the *p*-value of the test, (iii) for each remaining intron, we tested in the same way the GWAS3D scores of its variants against those of variants located in all other introns except the excluded one(s). This procedure was repeated until no significant signal was detected. The significant threshold was set at 1.92 × 10^−3^ corresponding to a Bonferroni correction for 26 tests for the 26 introns. Introns are then ranked by the *p*-value of the last Wilcoxon test performed with them. All statistical analyses were performed with the R software (version 3.6.1, R Core Team 2019).

### 4.2. Databases and URLs

Hi-C data track was obtained from human Embryonic Stem Cell line H1 (H1-hESC) and visualized using UCSC genome browser (http://genome.ucsc.edu, accessed on 7 May 2020). The hic contact matrix files and can be downloaded with the following accessions with this link (http://hgdownload.soe.ucsc.edu/gbdb/hg38/bbi/hic/, accessed on 7 October 2019) [62,65,66,67]. The degree of interaction is indicated by a more intense red color and is represented in red arc mode at a score maximum of 11 and a 1 kb resolution. DHS peaks from SAEC and Caco-2 cells were obtained and visualized using UCSC genome browser [46,47].

### 4.3. Culture Cells

Two cell lines were used epithelial adherent cells of intestinal origin from human colorectal adenocarcinoma (Caco-2) and pulmonary origin from human lung cancer (16HBE14o-). Caco-2 cells were maintained in Eagle’s Minimum Essential Medium (EMEM) supplemented with 20% Fetal Bovine Serum (FBS), and 16HBE14o-cells in Dulbecco’s Modified Eagle Medium DMEM supplemented with 10% FBS. Caco-2 and 16HBE14o-cell lines were grown at 37 °C in a 5% CO_2_ humidified atmosphere.

### 4.4. Plasmid Construction

All the cloning steps were done using the “In fusion^®^” strategy from Clontech. Using the pGL3-Basic Vector (Promega, Madison, Wisconsin, USA), the *CFTR* promoter (787 bp, “P*_CFTR_*”) was cloned upstream of the firefly luciferase cDNA, at the Hind III site. Candidate *cis*-regulatory regions and enhancer combinations were amplified and inserted downstream in the P*_CFTR_* construct (Appendix A, Table A1). Positive controls containing a fragment of −44 kb, intron 1 or 12 CREs of the *CFTR* gene were generated. All inserted fragments were verified by sequencing. Polymerase Chain Reaction (PCR) primers used to amplify the *CFTR* promoter, candidate enhancer sequences and control sequences are listed in Appendix A, Table A2.

### 4.5. Luciferase Assays

Cells (1.25 × 10^5^) were seeded in 12-well plates. Transfections were realized 24 h later with the transit 2020 reagent (Mirus, Madison, Wisconsin, USA). An amount of 400 ng of enhancers constructs and 100 ng of a pCMV-beta-galactosidase construct, as internal control, were used for each condition. Every condition was done in triplicate. At 48 h post-transfection, cells were washed once with 1X PBS (Phosphate Buffered Saline) and lysed with Passive lysis buffer (Promega). Cells lysates were clarified by centrifugation at 12,500× *g* for 4 min at 4 °C. A volume of 20 µL of each protein extract was used to assay the luciferase activity and 25 µL for beta-galactosidase activity. We used Promega reagents and a multiwell plate reader Varioscan (Thermo Fisher, Waltham, MA, USA). Results were presented as relative luciferase activity, with the P*_CFTR_* construct activity equal to 1. Significance of the increased luciferase activity was performed using unpaired *t*-tests using GraphPad Prism^®^ software.

### 4.6. Chromatin Immunoprecipitation and Polymerase Chain Reaction

Formaldehyde (Sigma, Saint-Louis, Missouri, USA) was added to 4 × 10^6^ Caco-2 cells (per immunoprecipitation) to a final concentration of 1%. Crosslinking was allowed to proceed for 10 min at room temperature and stopped by addition of glycine at a final concentration of 0.125 M. Fixed cells were washed and harvested with cold PBS. Chromatin was prepared following the SimpleChIP^®^ Plus Enzymatic Chromatin IP protocol (Cell Signaling Technology, Danvers, MA, USA). The Adaptive Focused AcousticsTM (AFA) Technology from Covaris was used in addition to enzymatic digestion with micrococcal nuclease (0.5 µL) in order to produce DNA fragments of 150 to 900 bp. Chromatin was precleared with protein G magnetic beads (Cell Signaling Technology) for 2 h at 4 °C and immunoprecipitation with HNF1α (#89670, Cell Signaling Technology), p300 (#54062, Cell Signaling Technology, Danvers, MA, USA), CDX2 (#12306, Cell Signaling Technology), TCF4 (#2569, Cell Signaling Technology), FOXA1 (#53528, Cell Signaling Technology), and FOXA2 (#8186, Cell Signaling Technology) specific antibodies, a negative control IgG antibody or a positive control Histone H3 antibody (Cell Signaling Technology) were carried out overnight at 4 °C. Immune complexes were recovered by adding protein G magnetic beads and incubated for 2 h at 4 °C. Beads were washed, DNA was eluted and cross-links were reversed following the manufacturer’s instructions. PCR were performed using HotStarTaq Mastermix (Qiagen, Hilden, Germany) and specific primers, as outlined Table A3. Different pairs of primers are designed to targets specific TFBSs according to prediction made by ContraV3. Several closely related TFBSs are detected by the same primer pair, as outlined in Appendix A, Table A3.

## Data Availability

Data is contained within the article.

## Appendix A

**Table A1:** Regions of *CFTR* candidate *cis*-regulatory elements cloned in pGL3-Basic Vector for luciferase reporter constructions.

Regions Name	Coordinates (hg38)	Size (pb)
Promoter *CFTR*	chr7: 117, 479, 274–117, 480, 060	787
−44 kb	chr7: 117, 434, 807–117, 436, 588	1782
Intron 1	chr7: 117, 489, 621–117, 490, 501	881
Intron 12	chr7: 117, 587, 777–117, 589, 290	1514
Intron 24	chr7: 117, 659, 283–117, 660, 272	990
Intron 26	chr7: 117, 665, 734–117, 666, 846	1113

**Table A2:** Primers used for luciferase reporter constructions.

Primers Name	Primers Sequence
pGL3B_BamHI_F	TTCAGGTTCAGGGGGAGGTG
pGL3B_BamHI_R	GTGCGGCGACGATAGTCATG
IF_BamHI_P*_CFTR_*_F	CGATCTAAGTAAGCTTGGAGTTCACTCACCTAAACCTGA
IF_BamHI_P*_CFTR_*_R	CCGGAATGCCAAGCTTTCTGGGCTCAAGCTCCTAATG
IF_BamHI_Int24_F	AAATCGATAAGGATCCGCATTTCTCACTCTGGCTGG
IF_BamHI_Int24_R	ATCGGTCGACGGATCCCCTGTCCAAACTAAGAAGACTTC
IF_BamHI_Int12_F	AAATCGATAAGGATCCTGGAGAAGGTGGAATCACACTG
IF_BamHI_Int12_R	ATCGGTCGACGGATCCGAAGACAGTATGCAAGAGCTACAT
IF_Int12-24_F	CTTGCATACTGTCTTCGCATTTCTCACTCTGGCTGG
IF_Int12-24_R	CCAGAGTGAGAAATGCGAAGACAGTATGCAAGAGCTACAT
IF_BamHI_Int1_F	AAATCGATAAGGATCCGTAGTGACATGCTCACTAATGC
IF_BamHI_Int1_R	ATCGGTCGACGGATCCCAGAGAAGGACGAAATTC
IF_Int1-Int24_F	ATTTCGTCCTTCTCTGGCATTTCTCACTCTGGCTGG
IF_Int1-Int24_R	CCAGAGTGAGAAATGCCAGAGAAGGACGAAATTCC
IF_Int1-Int12_F	ATTTCGTCCTTCTCTGTGGAGAAGGTGGAATCACACTG
IF_Int1-Int12_R	GATTCCACCTTCTCCACAGAGAAGGACGAAATTCC
IF_BamHI_Int26_F	AAATCGATAAGGATCCATCTGAGCCATGTGGTGAGGTTGA
IF_BamHI_Int26_R	ATCGGTCGACGGATCCGAAACTGGCACAGGCTCAAAGGAA
IF_Int12-26_F	CATACTGTCTTCATCTGAGCCATGTGGTGAGGTTGA
IF_Int12-26_R	CATGGCTCAGATGAAGACAGTATGCAAGAGCTACAT
IF_Int24-26_F	AGTTTGGACAGGATCTGAGCCATGTGGTGAGGTTGA
IF_Int24-26_R	CATGGCTCAGATCCTGTCCAAACTAAGAAGACTTC

**Table A3:** Primers used to analyse immunoprecipitation in Caco-2 cells.

Primers Name	Primers Sequence	Transcription Factors Specificity
ChIP_Int24_F1	GCACATGTGCTTTCTGTTCTGC	HNF1, p300
ChIP_Int24_R1	GATGGTAGAAGGAGGCAGTG	HNF1, p300
ChIP_Int26_F1	CCCTATGGTTTAGTCACAAGGAAGTT	HNF1, p300
ChIP_Int26_R1	GGCTCAAAAGCCTGAACAGAA	HNF1, p300
ChIP_Int24_F2	CAGCAGTTTGGGAAGTACATAC	FOXA1/2, CDX2, TCF4
ChIP_Int24_R2	CGTCCTGCCCCCAATATAATC	FOXA1/2, CDX2, TCF4
ChIP_Int26_F2	TCTAGCCATGATTTGGGTTC	FOXA1/2
ChIP_Int26_R2	GGCTCAAAAGCCTGAACAGAA	FOXA1/2
ChIP_Int26_F3	CATACAGCCATCCAGCTTTAC	TCF4
ChIP_Int26_R3	TCAACCTCACCACATGGCTC	TCF4
ChIP_Int26_F4	GAAGCAATGCTGGAATGCCAAC	CDX2
ChIP_Int26_R4	GTAAAGCTGGATGGCTGTATG	CDX2
